# Father George Ford, The Dominican Sisters of Sinsinawa, and the Catholic Progressive Corpus Christi School in Morningside Heights, New York City

**DOI:** 10.1177/01614681261438877

**Published:** 2026-04-05

**Authors:** Rosa Bruno-Jofré

**Affiliations:** 1 Queen’s University, Kingston, Kingston, ON, Canada

**Keywords:** Corpus Christi School, Teachers College, Father George Barry Ford, Catholic education, progressive Catholic education

## Abstract

The article examines the vision of education sustaining Corpus Christi School, located across from Teachers College in New York City, between the 1930s and early 1950s, and Father George B. Ford’s crucial partnership with the Dominican Sisters of Sinsinawa. The analysis is grounded in Quentin Skinner’s theory of intentionality and reference to context. It addresses the relationship of Corpus Christi School with Teachers College and Columbia University and the school’s distinctive features: its use of the project method, its social reconstructionist approach, and its way of “reading” John Dewey and William Kilpatrick. It discusses Ford’s theological vision inspired by early Christianity and his ecumenical position, as well as his intricate web of political, religious, and social relations. Also included is a social network analysis.

## Introduction

Father George Barry Ford (1885–1978), chaplain at Columbia University from 1929 to 1945, a crusader for human rights and labor rights, and a civil rights activist, headed Corpus Christi Parish from 1935 to 1958. Ford rebuilt the church and the school as a community center, which was inaugurated in 1936, and invited the Dominican Sisters of Sinsinawa to run the school, which was central to his ministry. This article examines the educational vision sustaining Corpus Christi School and its unique political positioning from the mid-1930s to the mid-1940s in light of Ford’s activism. Corpus Christi was a Catholic school located in Morningside Heights, across from Teachers College, in New York City. This neighborhood is on the Upper West Side of Manhattan bordering Manhattanville and Harlem; its parameters were placed in the 1930s between 110th and 125th Street. The school was often referred to as being avant-garde and progressive, forming part of a strand in American Catholicism that opened its own space in the configuration of educational ideas that developed starting at the end of the nineteenth century.

Many Catholic schools applied what is being called a moderate version of the progressive method with emphasis on active methods and child centeredness, and in the case of Dewey’s theories separating pragmatist philosophical tenets, in particular its naturalistic metaphysics and its attack on dualism. (I do not identify progressive education/new education, a global movement, with pragmatism.) Such was the case of Monsignor Bonner in the Archdiocese of Philadelphia, while the Dominican Sisters of Sinsinawa built a network of schools known for applying active methods within a depragmatized version of progressive education.^1^ However, a distinctive feature of the Corpus Christi School was not only its project method and its being child centered, but also its social reconstructionist approach, its way of “reading” John Dewey and William Kilpatrick, its close relationship with Teachers College and Columbia University, and Ford’s extensive connections with Protestant Social Gospelers, progressive Jewish rabbis, and intellectual and political leaders including Eleanor Roosevelt.^2^ However, Ford and the school encountered opposition from the hierarchy of the Catholic Church, which condemned modernity and did not agree with his political views. The Church has always been a plural institution, and although Neo-Scholasticism was dominant at the time, there were counter-currents including a preoccupation with social reform, such as the creation of *Commonweal* (a Catholic progressive magazine founded in 1924), which supported Corpus Christi School and the Catholic Workers movement.^3^ The Church was at the crossroads of Americanism and modernity, as is well illustrated by Corpus Christi School and Ford’s commitment to human rights and ecumenical organizations.

This paper provides a comprehensive picture of the Corpus Christi School, the role played by Father Ford in the school and beyond, and the work of the Dominican Sisters. It also addresses the school’s conception of education and its central pedagogical approach, referred to as “a new education in Catholic living.” I also discuss support for and opposition to the school. Although the school garnered a national and international reputation and had a presence in Catholic magazines and secular publications, it also received opposition and criticism from the Archdiocese of New York, and of course from the far-right, secular American Education Association. Ford interacted with the power structure of the Church at both the micro and macro levels while working within and intersecting with his own reformist vision and the liberal context of Columbia University.

In order to explore the school’s unique positioning, I trace the intricate web of political, religious, and social relations and networks, the kinds of connections generated by Ford and by the school itself, that extended beyond the Catholic Church and regular school connections. The school followed an overall social reconstructionist tradition that, with various nuances, thought of the school as building a new social order that was more just and humane, not revolutionary.^4^ However, from the 1920s in the United States, as Lawrence Cremin notes, the emphasis on child-centered pedagogy overshadowed concerns with social change, while in the 1930s, sectors in the profession sought to align progressive education with political progressivism.^5^ My analysis is grounded in Quentin Skinner’s theory of intentionality and his linguistic analysis, because the letters and documents written by Ford, the Sisters, and the life of the school and its curriculum can only be discerned in relation to often-conflicting contexts, such as the complex relationship between the New York Archdiocese and Ford, as well as the Sisters’ own lived experience.^6^ The paper focuses on the first ten years of Ford’s leadership in the parish and the school, extending from 1936 to the end of World War II. The post-war era opened a new contextual period. Ford was in charge of the parish and school until he retired in the spring of 1958, and the Dominican Sisters continued running the school for more than a decade afterwards.

One can only speculate as to why the history of Father Ford, the Dominican Sisters, and Corpus Christi School has not been written about more extensively, despite the school’s unique character resulting from Ford’s extensive progressive activist network, as well as the school’s relationship with Teachers College. It may be that Dewey’s specialists did not realize that Ford’s “reading” of Dewey and Kilpatrick was unusual for Catholics, who did not traditionally show a strong interest in Dewey. In fact, for Catholics, the Corpus Christi School and its methods were most certainly beyond the usual framework.

Perhaps another factor as to why the history of the Corpus Christi School has not been more fully examined by historians relates to the difficulty of accessing sources, in particular those related to the archdiocese of New York.^7^ The Dominican Sisters of Sinsinawa and their archivist were kind enough to provide all the documentation available about the school, as well as their own extensive correspondence with Ford and with their Superior. I also consulted the archive at Columbia University and Father Ford’s transcribed *Reminiscences* (1956) and his book *A Degree of Difference* (1969), the latter an autobiography; all these proved important sources.^8^

However, this paper is not without significant limitations. The documentation about Corpus Christi School is very incomplete. Furthermore, the Queen’s University archivist, librarians, and I were advised that the Archdiocese of New York archives do not hold either Ford’s papers and correspondence or the school’s records. As a result, I have thus far been unable to consult these missing sources.

## Corpus Christi School, Father George Barry Ford, and the Dominican Sisters of Sinsinawa: A Profile

George Barry Ford was born in Nashville, New York, 35 miles from Utica, where he was raised and exposed early on to an ecumenical world: listening to Protestant speakers and observing in the streets of his home city open ethnic and racial discrimination instilled in him a profound awareness of social injustice.^9^ Here is what he wrote in 1969 about his early assignment as a priest to St. Aloysius Church in Harlem, New York: “The Churches—both Protestant and Catholic—accommodated themselves to this social and political point of view [discrimination], instead of transforming it by living according to the precepts of the religions they preached. Their loyalty to the God of a Jim Crow church is one of the most widespread of modern blasphemies, and a glaring denial of the gospel of love and equality of persons.”^10^ In line with his ecumenism, Father Ford believed in the need to understand other religions and not just one’s own.^11^ He went well beyond mere tolerance and a scant interest in race relations, which was dominant at the time among so-called progressives.^12^

After serving as a chaplain in the army, Ford traveled extensively to the Holy Land and Asia. In 1929, Cardinal Hayes appointed him chaplain of the Newman Club at Columbia University, and in 1935 he became pastor of Corpus Christi Church at 535 West 121st Street, opposite Teachers College, in Morningside Heights, an area that soon became home for him. For the next ten years, Ford would hold two positions: pastor of the parish and counselor to Catholic students at Columbia University.

Ford selected the Dominican Sisters of Sinsinawa, Wisconsin, who were familiar with what has been referred to as Catholic progressive education, to run the school.^13^ This partnership, however, would not be free of tensions. Sister Joan Smith, OP, would play a central role in developing the Corpus Christi School curriculum, along with her thesis supervisor at Teachers College, Professor Roma Gans. Later, Sister Joan, together with Sister Mary Nona McGreal, OP, joined the Commission on American Citizenship. Formed in 1938 at the Catholic University of America in Washington, DC, the Commission emerged as a response to Pope Pius XI’s call to develop “a constructive program of social action” that could apply “principles of justice and charity,”^14^ a position situating the Church in relation to American modernity without challenging the social order or the Neo-Scholastic framework of the Church, but in line with social encyclicals. Under the Commission’s mandate, the two Dominican Sisters co-authored a three-volume work, *Guiding Growth in Christian Social Living*, published in 1944–45, which was used at one point at Corpus Christi School to teach religion.^15^

Why the Dominican Sisters? The Congregation traces its history to the thirteenth century, when Dominic founded the Order of the Preachers (OP). The US Congregation was founded in Wisconsin by Italian Dominican missionary Samuel Mazzuchelli, OP, in 1847. The Sisters from Sinsinawa are “called to proclaim the Gospel through the ministry of preaching and teaching in order to participate in the building of a holy and just Church and society.”^16^ Ford was clear: he wanted teachers who had received advanced pedagogical training in secular colleges and universities instead of those trained in Catholic colleges who had acquired only a “knowledge of a traditional and outmoded routine.”^17^ Those teachers attending Catholic colleges, in his view, had not been exposed to modern pedagogical methods, had learned to teach by rote, and adhered to the diocesan plan for education.^18^ The Dominican Sisters of Sinsinawa, who had earned degrees in Wisconsin’s state universities and colleges, and many of whom were studying at Teachers College, would, in Ford’s estimation, form the ideal team of teachers. Furthermore, the Dominican Sisters had a network of schools in which they used active methods, and they described their pedagogy as child-centered within the framework of Catholic education as it was being developed in US society at the time.

The Corpus Christi School complied with the general features of progressive education, as outlined by Cremin: a broadening of the school that would include a concern with health, student vocation, and the quality of the community; pedagogical techniques deriving from research in various disciplines, in particular psychology; tailoring instruction to children; and the use of more systematic and rational approaches to the administration of the school.^19^ Nonetheless, as I will show, Ford’s vision went beyond child-centeredness; his social vision was daring, as was his idea of school as a transformational agency.

Ford discussed his projected school with a number of Sisters: Sister Antonine Goodchild, who was completing her master’s degree at Teachers College; the newly appointed principal of the school and superior of the Convent, Sister Vivian Doran, who was completing her master’s degree at Columbia; Sister Mary Charles Schlenk, who was completing her doctoral dissertation on education and was interested in the progress of the school and its curriculum; and Sister Joan Smith and her supervisor at Teachers College, Roma Gans, who would play a central role in the design of the school curricula.

## The Setting

Corpus Christi Church and school were located in Morningside Heights on West 121st Street in a culturally diverse and heavily populated area comprising Columbia University, Teachers College, Barnard College, Union Theological Seminary, International House, Jewish Theological Seminary, the Cathedral of St. John the Divine, Riverside Church, Corpus Christi Church, and the Juilliard School of Music.^20^ Ford enjoyed cordial and friendly relations with all the leaders of these institutions. Roma Ganz, a Teachers College specialist in reading, wrote that although about two-thirds of the population was native-born white, and the majority were Irish, there were many other nationalities in the neighborhood. She assessed that most families in the parish were poor and lived in crowded conditions in apartment houses with poor lighting and ventilation. The streets were closed to traffic to allow children to have a space to play, the low-crime neighborhood also had limited cases of juvenile delinquency and few divorces, and the moral tone of family life was generally high.^21^ In 1936, during the Great Depression, the General Superior of the Congregation, Mother Samuel Coughlin, described the children as belonging “to a very poor class, many of their families are on relief and some of them have probably not enough to eat.”^22^ In 1930 the area was by and large white and middle-to-upper class, with a few African Americans, but the Depression caused the demographics of the area to change, particularly with the subdivision of apartments and increasing single room occupancy, while being mostly a white neighborhood.^23^

## The New Social Complex and School

The old building of Corpus Christi Parish included a Catholic school with the same name as the Church. In fact, the entire structure was, in Ford’s view, unsuited for the purposes he had in mind. The school had been closed for a year when reconstruction began. Ford wanted a Colonial American–looking church and hired architect Wilfred E. Anthony, formerly associated with Gothicist Ralph Adam Crom, to design a new building.^24^

Ford submitted the proposal for a new building to the congregation, something very unusual at the time. From the outset, he envisioned the parish as an irradiating cultural, spiritual, and social center that would include the church but also facilities such an auditorium, venues for athletic activities, moving pictures, plays, parties, the convent, and the school. The entire complex was aimed at integrating the community with the school and the church proper, which would be open to all, not only to members of the congregation. The building was eight stories high. The church on the first floor occupied a story and a half and the school occupied three floors; the top floor was a convent, and beneath the church was a large multipurpose auditorium. There would be religious services, religious societies would hold activities, and there would be forums and lectures about a variety of issues and current political and social problems.^25^

Classes began on September 14, 1936, and the building was officially dedicated on October 25 by Cardinal Hayes in the presence of Dr. Nicholas Murray Butler (president of Columbia University from 1902 to 1945), sixty faculty members from Columbia (including deans), and representatives of several colleges in the city, among them Carl W. Ackerman, president of the Columbia School of Journalism, and Virginia Gildersleeve, dean of Barnard College.^26^

## The School

The school had an enrollment of 500 students, with about thirty students in each class from kindergarten to eighth grade.^27^ The design of both the space and the furniture highlighted a shift in the materiality of schooling in line with the school’s avowed progressive education and its use of the project method. The space in the new building was planned and designed by Dr. Nickolaus Engelhardt, who had previously planned large schools in the country. Roma Gans, professor of elementary education at Teachers College, designed the school’s furniture, which included portable desks of various heights to fit the size of each child, and chairs made of light maple, making them very easy to move in order to generate a cooperative environment.^28^ The walls in each room had a different color, and these soft, pleasing colors differed markedly from the monotonous colors of traditional schools.^29^ Other equipment was planned under the direction of Florence Stratemeyer, a specialist in elementary-level curriculum at Teachers College.^30^

The goal at the school was to create an interplay with material practices that would generate a more participatory, democratic pedagogy. The setting would provide students with the “freedom to move about, to talk freely, to express opinions, and make decisions.”^31^ Dominican Sister Joan Smith described the school as “decidedly avant garde! In fact, revolutionary for its time. What was traditional was ‘out’; what was progressive or innovative was the thing.”^32^

Not all the Sisters were so positive, however. There was even a concern that the school could “lean toward ultra modernism in procedure.” In fact, principal Sister Vivian, writing to Mother Samuel, stressed that “the first purpose will always be to train those children to love the teaching of Christ, both through word and example.” As she explained, “[A]n activity program does not mean a wild departure from the formal teaching but rather creating living experiences, the aim being to intensify interest in and knowledge of the subject matter.”^33^ She told her Superior not to have undue fears,^34^ which were quite understandable, as from 1910 until the 1960s the Vatican required from preachers, teachers, pastors, and professors an anti-modernist oath, although it does not seem to have been imposed in the school.^35^

“New Education in Catholic Living” would be the Corpus Christi School program’s slogan for some time. The original vision of the school was intended to embody a pedagogical progressive strand rather than an administrative one, the latter more concerned with efficiency, although the sparse documentation available would also suggest, at some points, the use of an eclectic approach.^36^

Father Ford’s correspondence shows that he played a central role in selecting the Dominican Sisters and in assessing their performance regularly.^37^ This proved difficult for some of the Sisters, because many had prior experience practicing active methods in progressive education schools. Ford’s style with the Sisters was a complex refraction of the gendered character of the Church. Some of Ford’s letters to the Superior of the Congregation questioned the suitability of specific Sisters to teach in what Ford called “a set-up in many ways different from the traditional procedure.”^38^ He wrote that “we all feel here that we have a powerful faculty in the school. But our third grade is noticeably weak.”^39^ There are letters of great praise of the Sisters as well, but from the beginning to the end of Ford’s leadership, there are also many letters expressing concerns with Sister-teachers and the possibility that they could try a hybrid model.^40^ There are also letters from Sisters themselves expressing their distress to the Superior General over Ford’s complaints.^41^ In 1943, for example, Sister Bernadetta wrote: “We are being inspected and supervised *very frequently* these days, by FF. He will have no semblance of traditional procedure and whenever he comes upon it in a room he immediately takes action.”^42^ The General Superior, in turn, expressed concerns with the amount of work the Sisters were given:

They have, in a certain sense, at least twice or even three times the duty of a teacher in a formal school: first, they must follow the progressive program with its projects and activities, well planned from day to day; they must make sure of giving the pupils a thorough training in the fundamentals, which require at least a minimum of drill, and which are given most of the time in formal schools of the archdiocese, and with these formal schools they must take the diocesan examinations. Lastly, there is a certain amount of strain attached to the presence of visitors at any time that they will come.^43^

Indeed, in April 1947, Ford wrote: “this year alone nearly 500 educators have visited our school, and more and more is it referred to in all parts of the country according to reports and comments that are received and heard.”^44^ By June 1947, the number of visitors had reached 700.

Changes in the teaching body were a recurrent problem in the school. They were due either to requests from the teaching Sisters, to other obligations, or to questioning by Ford,^45^ who made it clear that the main problem resided in teacher preparation: “We find not a few situations in our school that really are contradictory of the original intent, the purpose, and the accomplishments theretofore so admiringly recognized. . .. One of our problems has been to reeducate in the new methods of education some of the teachers assigned here.”^46^

## What Was New Education for Catholic Living?

An important feature of the school was the relationship of education to the Catholic philosophy of life. The latter was concerned with “the whole child, [who] is ever conscious of his nature and his ultimate destiny as well as of the importance of his moment by moment living and experiencing. It takes account of the varied relationships which affect him—relationship with himself, with his fellow men, with the world about him, with his Creator. . .. And Catholic education functions to develop well integrated personalities, adjusted to living an abundant life in an ever-changing civilization.”^47^ There was an obvious separation between nature and culture. An interesting feature is how the school’s pedagogical approach integrated Dewey’s concepts, such as critical inquiry, the notion of education as having both a psychological and social dimension, and notions of growth, among others, without embracing or rejecting a naturalistic, pragmatist metaphysics.

The school relied on the project method, in this case a mediated Deweyan philosophy of education without reference to pragmatism, and regular allusions to the project method were made without attribution. The Vatican did not accept pragmatism as a philosophy, and of course John Dewey’s work was usually “de-pragmatized” to incorporate Dewey’s concepts into Catholic education.^48^ Ford was on good terms with William Kilpatrick, who, in Ford’s words, helped him “to understand the greatness of John Dewey.”^49^ It is safe to say that Ford, in light of his own references to Dewey, not only met him, but was also very familiar with his work.

**Figure 1.**
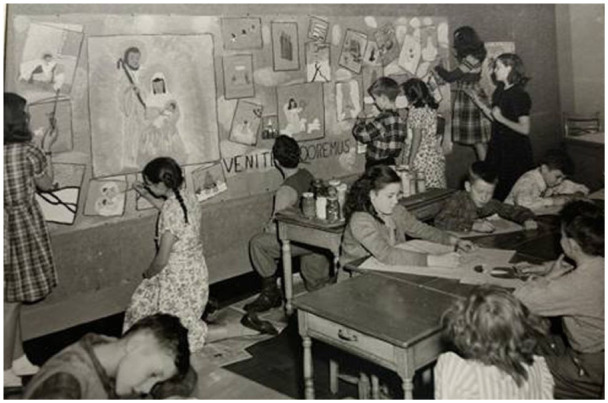
A photograph from Album 1948, Corpus Christi School, placed in the rectory of the parish. In October 2024, the parish priest, Father Peter Heasly, provided the author access to artifacts to be used in research work.

Experiencing and living, growth, and cooperation were central concepts in the classroom, as was problem resolution (in line with Dewey’s theory of inquiry). The school emphasized the social nature of the activity method.^50^ The teacher played an important role, while student-centered and creative expression did not mean unlimited freedom. Growth was understood as being concerned with understanding, Roma Gans wrote, rather than with subject matter, an approach related to the notion of socially constructive living through responsibilities given to students in running school activities. At Corpus Christi, the “Tentative Plan for Guiding the Growth of Children” described individual growth in harmony with the progress of the group while cultivating self-direction; growth progress consisted of developing a social consciousness that recognized the interdependence of the individual and the group. As with Dewey, the concept of growth at Corpus Christi appeared to be related to an understanding of democratic life. However, Dewey’s naturalistic notion of growth with no fixed end—growth in education would be more education—and no end beyond itself, although Dewey had social goals, appeared to be mediated by a Catholic philosophy of education that sought a useful Christian life in this world and in eternity a perfect union with God (Figure 1).

Moreover, the school was concerned with the whole child, another trait of progressive education. Moral integrity, not far from Dewey’s position in his *Democracy and Education*,^51^ would require standards of action that could be applied to all activities of life.^52^ Cooperation instead of competitive individualism led to group inquiry and enterprises; social relations were at the core of learning, and learning was conceived as a social experience. Roma Gans wrote, “This is in line with the theory of the school: To provide an environment which helps the child to learn to solve his problem so that he may establish the necessary and desirable controls for effective living through (a) increased sensitivity to problems, (b) power to work out a plan of action to deal with the recognized problems, (c) needed tools to carry the plan into action, (d) willingness to execute the plan.”^53^ All this was in line with Dewey’s theory of inquiry.

The teacher played an important role in this pedagogical approach, one in which the curriculum organization based on subjects was replaced with one based on units of activities. The pedagogical principles of the school were centered in an inquiry—central to democracy, as in Dewey—that would be balanced, following Gans and the Plan for the School, with an understanding and appreciation of the doctrines and practices of faith; of beauty found in the arts, nature, and human conduct; of the problems of others; of the social and industrial phases of life; of the interdependence of individuals and peoples; and of the continuity of human progress.^54^

The children at the Corpus Christi School analyzed problems affecting the community and workers, weather conditions, adequate heating of homes, and dressing for cold weather. Gans quotes from the School Plan: “Learning is the progressive modification of behavior brought about by the interaction of the individual and his [sic] environment.” This was all very Deweyan. The children participated in the planning of the day.^55^

In a 1943 article in the *Journal of Educational Sociology*, the school program was described as an outstanding one in terms of social responsibility and Americanism. The article described how eighth-graders examined their own prejudices in order to develop open-mindedness and critical thinking, and to protect a democracy that respected difference.^56^ In addition, to complement their curriculum, Corpus Christi invited specialists in science, art, music, and in understanding foreign cultures, and both children and teachers sought contacts with foreign countries, members of other school faculties, parents, and children from other schools. Groups of children from other schools also came to Corpus Christi to demonstrate their talents and share their discoveries.^57^ At Corpus Christi, report cards were replaced by teacher–parent conferences; the school had a student council, a bank, a newspaper, and even a student-run store.^58^

One interesting, yet unexplained, point is fallibility linked to an inquiry process, because the Catholic doctrine had to be integrated with the program provided by the archdiocese. Ford may have been inspired by Dewey’s philosophy, but the issue of faith had to be articulated in some way. Dewey did not see the fundamental purpose of inquiry to be the pursuit of certainty, but a more provisional “warranted assertability.”^59^ Father Ford and the Sisters did not work out potential theoretical contradictions; in fact, although critics and supporters linked the school to Dewey’s philosophy, they remained mute about their sources. There was definitely a close relationship with Teachers College and an affinity with social reconstruction, but there are no theological references in the documentation at a time when the *nouvelle théologie* and its engagement with modernity was gaining a space—albeit outside the Vatican walls. There is a peculiar silence broken by active involvement in human rights and ecumenical committees and the Sisters who obviously translated views into practice.

A number of Catholic schools used activity methods, but Ford and the Sisters went beyond that. The school had an ethically grounded vision of education—which was not within the parameters of the Vatican and its Neo-Scholasticism—and its notion of how to live the faith. The school pedagogy cultivated social justice and fought racism within an open pedagogical process grounded in a reformist approach in the progressive liberal tradition and in the conciliation of classes. This pedagogy could be understood as being in line (albeit mediated) with the social reconstructionism that had been developed at Teachers College around the study group of William H. Kilpatrick, R. Bruce Raup, George Counts, Harold Rugg, and Goodwin Watson, all of whom met to examine the impact of social change on education. In fact, in 1934, the group founded *The Social Frontier: A Journal of Educational Criticism and Social Reconstruction*, which, at the time, was considered radical in its approach to education as social reconstruction and its view of teachers as agents of social change.^60^ There is no mention of *The Social Frontier* or of its leaders in the documentation related to Corpus Christi School, or of the Progressive Education Association, which took over the journal in 1939 but languished after the war.^61^

In his 1956 *Reminiscences*, Ford said that he had a persistent opposition to the “pinkos.” This statement makes sense in the context of the encyclical *In Divini Redemptoris*, issued in 1937 by the Vatican, that denounced Communism, making the anti-communist struggle paramount and the focus of the Cold War in the 1950s.^62^ It is not clear what Ford exactly meant by “pinkos,” because he also supported socialist ideals during the Great Depression, asserting that “I love a liberal position.”^63^ Ford’s statement is understandable, however, because Catholics had been seen as suspicious ever since the Red Scare of the 1920s, linking them to totalitarian thinking in the context of anti-Catholic feelings.^64^

In any case, Ford was clear that the Church’s hierarchy, particularly in New York, considered as “pinko” or communist any position contradicting the Church’s official stance. In his *Reminiscences*, Ford states that he leaned very strongly toward the radicalism that existed in the early thirties on campuses, particularly among students (at Columbia University, for example).^65^ He also said that his social vision had been inspired by Leo XIII’s 1891 encyclical *Rerum Novarum* and Pius XI’s 1931 encyclical *Quadragesimo Anno*.^66^ The first was a response to the social problems of industrialization and the right of labor to organize, while it aimed at constructing a corporativist Christian social order as an alternative to liberalism and socialism.^67^ The second, addressing the ethical implications of the socioeconomic order, attempted to clarify the rights and mutual duties of the poor and wealthy, of labor and capital.^68^ These documents delineated the social teachings of the Church, which were grounded in a concern with the common good, challenging both Marxism and capitalism.^69^ These encyclicals also generated Catholic social and political action and the engagement of young people, something not always welcomed by Church authorities.

In the 1930s, the institutional Church was engaged in what James Chappel called “paternal Catholic modernism,” with family and reproduction at the center but with little commitment to civil rights, democracy, or anti-racism.^70^ On the other hand, the racial issue was very important to Ford, and he involved his Newman Clubs in working at a center in Harlem.^71^ In a 1936 letter, Ford wrote that “All people are our brothers no matter what color their skin may be,”^72^ and in 1942, he said that there was no problem more pressing in the United States than the color question: “While the United States is dedicated to democracy as a way of life and while we are fighting fascism abroad we are denying to the largest minority group in the USA—fourteen million negro Americans—the privileges which are their rights as citizens in a democratic society. . .. [B]ut thanks be to God it is making many of the white population, both as democrats and as Christians, ashamed in the presence of this obvious hypocrisy.”^73^ In his letter, he asked the Dominican Sisters to provide teachers for a parish in Harlem, run by Father O’Sullivan, which was “exclusively colored.”^74^ Unfortunately, given their many current obligations, the Sisters could not comply. Ford wrote that when the world settles down, “the underprivileged groups in the United States are to be the predominant concern of government, of socially minded individuals, and certainly ought to be of the Church.”^75^ Ford referred to the US system erecting “concentration camps against many of its own citizenry in nearly every community in the country.”^76^ He expected the Church to act as a Christian body to denounce this situation.

## Opposition and Support

Ford wrote to Mother Samuel, Superior of the Congregation: “. . . I by no means stand-alone even though unwarranted restrictions have been placed upon me—denying my rights as a priest and citizen. . .. Some day the Holy Spirit may return to New York.”^77^ In the early days of his tenure at Corpus Christi, Ford’s conflicts with the archdiocese took a personal turn and resulted in prohibitions placed on him in addition to criticism of or impositions on the school. He had run-ins with Chancellor (later Cardinal) Msgr James Francis McIntyre, and with Archbishop (1939)—and later (1946) Cardinal—Francis Spellman.^78^ Both were very right wing and adhered to the Vatican’s most conservative positions. A powerful testimony of Ford’s resistance to the hierarchy and of his adhering to his convictions was his letter to McIntyre in response to the Chancellor’s request that Ford withdraw his sponsorship of the National Committee to Combat Anti-Semitism and its proposed publication *Counterattack.* The reason, Ford argued, was associated with the progressive names of congresspeople on the Committee.^79^

Chancellor McIntyre had significant difficulties with Corpus Christi School and with Ford himself, and these increased when, in 1937, the Corpus Christi Parish monthly magazine *Chronicle* denounced two well-known firms for underpaying employees. Moreover, Ford allowed some 500 Consolidated Edison employees to use the school auditorium to discuss their wage demands. For Corpus Christi, this was a matter of social justice, while for the Chancery, it underscored the very real fear that the company would cut contributions to Catholic charities.^80^

The diocese conveyed its opposition to Ford’s pastoral and educational work in several ways. The *Chronicle* was questioned because of its outspokenness, while the parish was forbidden by the Diocesan Council to have the compline in English in the novenas, which intended to promote the participation of the laity. (Prayers and petitions were allowed in the vernacular.) A special point of friction with the archdiocese was that the children’s mass, the Mass of the Angels, be sung one Sunday each month by the children. One Sunday the children had a Misa Recitative, and on two Sundays they said their own prayers. In a letter to Mother Samuel, Ford wrote that the school, the Church services with lay participation, and the *Chronicle* and their discussions were “all receiving unjust condemnation.”^81^ The school, he wrote, would revise the curriculum to follow the Regents and the diocesan syllabus, but Ford resisted closing the school to visitors because “it would do irreparable harm to the church in this country.”^82^ Toward the end of his letter, in which he related to Mother Samuel the critical points from the Church authorities, he wrote: “We can still do our job here, pay tribute to the diocesan system many parts of which we do not like and which are educationally unhealthy, follow the requirements of the Regents . . . and [we] still retain our valuable methods.”^83^ Ford referred to the memory-based exercises and unquestionable truths. Mother Samuel’s reply reflected the official position of women in the Church: “The application of the letter to us [referring to the questioning by Church authorities raised by Ford] is difficult to understand because we religious communities of women teach in parochial schools under the direction of the pastors and do not inaugurate new systems of teaching or other innovations, except under the direction of the pastors.”^84^ Ford responded with two letters. In the first, he made it clear to Mother Samuel that the school had the knowledge of and approval by the archdiocese superintendent of Catholic schools, who in turn had permission from the Vicar General of the Diocese and head of the Diocesan Council. The letter from the Council questioning the use of progressive methods not only pointed to Corpus Christi: “this letter applies to the progressive group within each community that is becoming publicly critical and they [the New York diocese] are using the authority of the diocese to stifle it. Of course, it is silenced but not killed and only for a time.”^85^ In the second letter, Ford asserts his authority as per Canon Law as a pastor with authority over all parish activities. He makes it clear that he had made all the decisions in relation to the construction of Corpus Christi School, its design, methods of education, furniture, and teaching staff, without consulting the community of Sisters, and that the pedagogy was followed at his insistence. Consequently, any criticism against the Sisters’ community was misplaced.^86^

The following paragraph in another letter conveys the character of Ford’s relationship with the Church authorities:

In dealing with a few close to the center of authority in the New York Archdiocese, it is distressing, if not alarming, to observe that they are literally fiddling while Rome is burning. Preoccupied with minutiae, imperative needs and issues remain unseen and, of course, unsolved. Inaction invites approval. Any understanding that diverges in the slightest degree with what has been and from the small thinking of those in authority, provokes disapproval and persecution. The good Lord alone can untangle much confusion in their minds and give them the light to see these needs and opportunities that unseen are holding back and are weakening the Church more than attacks of any enemy.^87^

Ford saw the school as a “most alluring opportunity for the Church in America.”^88^

In 1944, Ford published an article about Corpus Christi in *Catholic Religious Education*, placing his experimental school within the Encyclical Letter on the Christian Education issued by Pope Pius XI. Ford’s words can be construed as a response to his critics:

Christian education takes in the whole aggregate of human life, physical and spiritual, intellectual and moral, individual, domestic and social, not with a view of reducing it in any way, but in order to elevate, regulate and perfect it, in accordance with the example of the teaching of Christ. The development of this purpose in Catholic education demands a conscious interrelationship between home, school, and church, where each assumes definite obligations and responsibilities for the development of the child. In the fulfilment of its obligations Corpus Christi is striving to offer to its children the best possible kind of education for life in the modern world.^89^

As a mediator, Ford gave a recontextualized meaning to his reading of the encyclical. He moved away from the conventional Catholic meaning coming from the Holy See regarding the interrelation between home and school, one that officially stated that “education belongs pre-eminently to the Church, by reason of a double title in the supernatural order.”^90^ Ford was trying to solve a problem generated by the dogmatic Neo-Scholastic approach of the Vatican at the time, one that rejected Dewey’s ideas and in particular his pragmatism. He actually put in practice a new understanding of Catholic education, a concept of holistic education that, while keeping some features of conventional normative meaning, was removed from the framework required by the Church.^91^

The school also received support from important sectors of the Church. Thus, the superintendent of schools in the diocese of St. Augustine requested a picture of the school to show to teachers in the diocesan system; Msgr Johnson in Washington had a picture there to show to educators in that city.^92^ Meanwhile, *Commonweal*, the Catholic progressive magazine run by lay people, targeted readers willing to engage with the Catholic tradition in the context of justice and critical intelligence. It published an invited article about Corpus Christi by Frances G. Sweeney, a teacher at Lincoln School in New York City that was attached to Teachers College. The article, “A Catholic Progressive School,”^93^ described the school and its workings, informing readers that the fifth-graders studied Jesus directly from the New Testament and that the children participated in High Mass. Sweeney also countered the usual criticism of presentism that one of the basic aims was “the transmission of the best cultural heritage of the past to the young generation.”^94^ In order to make her point that the school worked well, Sweeney mentioned standardized testing, writing that the Modern School Achievement Test showed grade six for the school year 1936–37 to be above the median for the grade in reading comprehension, reading speed, and spelling. In arithmetical reasoning, computation, and language usage, however, the grade was below the median. In May, the total achievement median had risen from 6.2 to 6.8 and the only median below the grade level was arithmetical reasoning, which had risen from 5.7 to 6.1.^95^ Sweeney also made an important point when she mentioned that there was no selection of students based on abilities, reiterating the principle that students were expected to succeed in social situations as well as in academic pursuits.^96^ Sweeney’s closing paragraph placed the school within the liberal reconstructionist strand mediated by Catholicism, as understood by Ford, and in no small way in accordance with Dewey’s notion of democracy: “The best training for living in a democracy comes through living in a democratic way,” she wrote. “The school that can furnish opportunities for children to think for themselves, train them in working for the common good of society, and inculcate in them ideals of practical Christian life has done no small part in helping to develop good citizens.”^97^

Widening the discussion regarding Corpus Christi’s approach to Catholic education, another writer, Clara C. Glenn, responded to Sweeney’s *Commonweal* article questioning the notion that education is concerned with the development of the whole child, because in her view the business of the school should be the development of the intellectual power of children, not of all their powers. She attacked the stress on the problem-solving of current issues, her perception of a neglect in transmitting cultural heritage, the design of the curriculum that broke the separation of categories of learning, and the emphasis on experience, while overlooking skills such as good writing.^98^ Finally, Sister Mary de Lourdes, intervening in the conversation conducted in *Commonweal*, defended the principles of progressive education and the formation of the child as a Catholic agent of change “cooperating with God in perfecting the world.”^99^

In 1937, *The Curved Horn*, a periodical publication of Teachers College, Fordham University, New York, also published an article on Corpus Christi School, giving a positive assessment of how the project method worked in the school while praising the disciplined cooperation and work of the Sisters.^100^ Other Catholic publications also provided positive analyses.^101^ It is not surprising that *The Educational Sign Post for Better Schools*, the newsletter of the local right-wing teachers’ association called the American Education Association (AEA), also published an article about Corpus Christi School, pointing out that progressive education, as conceived by the John Dewey Society, the Social Frontier thinkers of Teachers College, and the leaders of the Progressive Association, was a menace to religion and, moreover, too close to Russia in their approach. The AEA advised Corpus Christi to become familiar with the system of Antonio Rosmini Serbate, a nineteenth-century Catholic philosopher.^102^

## The Social Network, Father Ford, and Corpus Christi School

**Figure 2.**
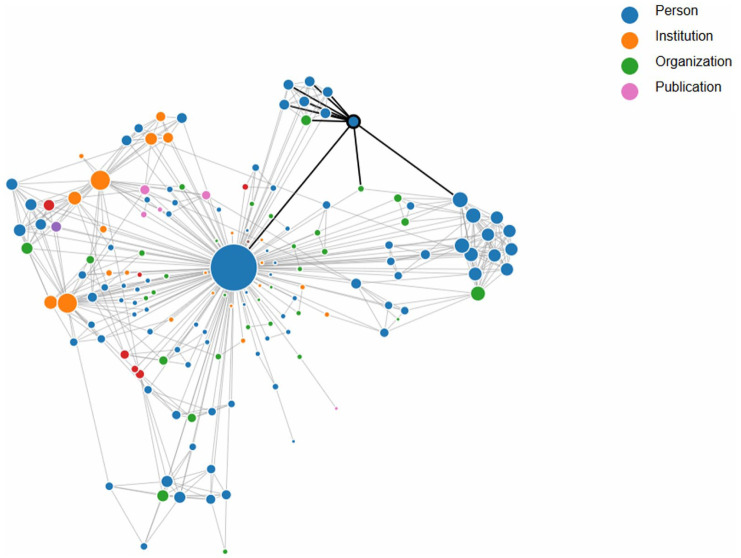
The network of people, institutions, organizations, and publications with which Father Ford was involved. Ford is represented by the large blue circle (node) at the center. The nodes are sized according to degree; in other words, the largest nodes have the largest number of connections. The largest two institutions represented here in orange are Columbia University and Corpus Christi School. This network visualization was created by Dr. Ana Jofre, SUNY Polytechnic Institute, Utica, New York. An interactive version is available at https://theirgroup.org/FatherGeorgeBarryFord/.

The social network and Ford’s politics are reflected in the school’s ethos, evincing, firstly, a close relationship with Teachers College and Columbia University at large; it had its roots in Ford’s involvement as a chaplain of the Newman Clubs and of Catholic students, and in the role played by Teachers College in being a site of new conceptions of education. Secondly, the network had an ecumenical dimension in line with Ford’s ecumenical view of religion, thirty years prior to Vatican II. Thirdly, it had a strong active political dimension, conveyed in the many commissions in which Ford was involved, such as the Freedom House, the State Department Committee for Foreign Students, and the engagement of the school with the United States Office of Education. Fourthly, it had a social dimension that included Ford’s engagement with civil rights, labor issues, interculturality, and human rights. The full network of people, organizations, institutions, and related publications surrounding Ford is illustrated in the interactive visualization in Figure 2.

From the archival documentation, it is clear that Ford nurtured a close relationship between Teachers College and Corpus Christi School, in which the Sisters played a central role in supervising student teachers, observing classes, practicing teaching, and hosting visiting professors. For his part, Ford lectured in classes at Teachers College, while Columbia professors made presentations to parents at Corpus Christi.^103^ From the outset, the Superior of the Dominican Sisters made it clear to her Sisters that “Father [Ford] expects that the Sisters welcome visits from members of the faculty of Teachers College.”^104^

During his tenure at Corpus Christi, Ford collaborated on numerous occasions with William Kilpatrick (known for the project method) regarding both pedagogical matters and human rights issues. The interaction with various colleges and departments—including not only faculty and student teachers teaching music and art, and directing testing programs at Corpus Christi, but also the provision of medical examination of children, dental services, cataloging in the library, and speech examination, among others—also indicates a regular and broad interaction with the university at large. Two Catholic faculty members at Teachers College, Roma Gans and Florence Stratemeyer, worked with the Dominican Sisters in developing the curriculum at Corpus Christi. There was also a regular connection with Dean Russell of Teachers College, and Ford was close friends with Nicholas Murray Butler, the President of Columbia University. Responding to many requests, Ford and the Sisters opened Corpus Christi School to a large number of students attending the summer session at Columbia. Many were Sisters, Brothers, and lay people (Catholics, Protestants, or nonbelievers). These visitors posed many questions about the newer education methods being implemented at Corpus Christi.^105^

There was also an interesting intersection of Columbia professors with chaplains, and with Ford’s participation on boards and committees, which showed political links between a progressive conception of education, including progressive Catholic education and human rights, and ecumenical organizations pursuing related agendas dealing with the causes of racial and religious prejudice and social problems. In 1928, human rights activist Dr. Everett Clinchy, reacting to anti-Catholic bigotry, founded the National Conference on Christians and Jews. Ford sat at many meetings with Newton D. Baker, the former US Secretary of War (1916–21) representing Protestant churches, industrialist Roger Williams Straus representing Jews, and Professor Carlton J. Hayes of Columbia University representing Catholics.^106^

In 1944, Ford and Sister Mary of Lourdes of St. Joseph College in Hartford, Connecticut, were Catholic representatives on the board of the Service Bureau of Intercultural Education. The Bureau, formed in 1934 by human rights activist Rachel Davis DuBois, who obtained her master’s degree from Teachers College, was chaired from 1938 by William H. Kilpatrick from Teachers College (prior to which the executive had been chaired by Heber Harper from Teachers College). The Bureau incorporated the Education Division chaired by Professor H. H. Giles, a friend of Ford from Columbia University.^107^ It produced curriculum units and books on intercultural education that were used at Corpus Christi, as well as at other schools wishing to incorporate more progressive, democratic ideals.^108^ The Progressive Education Association also provided some funding for the Bureau’s efforts. Connecting to concrete situations, Ford’s preoccupation with racial discrimination and segregation led him to serve as a member of the executive of the Citywide Citizens’ Committee on Harlem to alleviate the poor living conditions there. Formed in 1941, the Citywide Citizens’ Committee on Harlem had various subcommittees dedicated to housing, health, education, employment, and mobilized public opinion to put pressure on influential people and groups.^109^

Ford was also a member of the Church Peace Union, founded by Andrew Carnegie, which included Protestants, Catholics, and Jews.^110^ In that capacity, Ford delivered an address at a Mass that closed the first day of the 1947 Institute of Religion at the United Nations and the thirty-second annual meeting of the World Alliance for International Friendship Through the Churches. In his address, Ford chastised Cardinal Spellman and Methodist Bishop Bromley Oxnam for name calling, and also chastised Catholic Archbishop Richard Cushing for not being able to discuss issues calmly. The incident was published in the *New York Times*, and Spellman prohibited Ford from ever speaking in public again.^111^

Father Ford’s ecumenism was also reflected in his close relationship with Union Theological Seminary (interdenominational, Protestant, progressive Social Gospelers) and with Dr. Harry Emerson Fosdick, who presided over Riverside Church (Protestant).^112^ He spoke annually at Union Theological Seminary luncheons and attended, with Rev. Dr. Alexander Griswold Cummins, the Annual Synod of the Episcopal Diocese of New York. Also noteworthy are Ford’s friendship with John Haynes Holmes, founder and pastor of the Community Church in New York, who collaborated with Rabbi Stephen Wise (a friend of Ford), and his association with Rabbi Jacob Weinstein, counselor of Jewish students at Columbia. Ford also collaborated on the television program *Crossroads*, which aired on ABC from 1955 to 1957 and was co-created and co-sponsored by Ford, alongside Rabbi William F. Rosenblum of Temple Israel, New York, a leader of Reform Judaism, and USN Captain Maurice M. Witherspoon, a Presbyterian Minister and the vice president of the Military Chaplains Association. The program anthologized case studies of spiritual advisors’ experiences with people dealing with various societal problems.^113^

Ford’s political connections were important. He was very close to Herbert B. Swope (1882–1958), executive editor of the *New York World* and during World War II a consultant to the US secretary of war. During the war, Ford was also a member of a State Department committee^114^ and was close friends with Eleanor Roosevelt. They worked together at Freedom House (created in 1941 to counter Nazism), an organization committed to democratic principles. In 1953, Ford was selected by the American Committee on Intellectual Exchange to go to Japan, and Eleanor Roosevelt accompanied him. It is not surprising that the United States Office of Education selected Corpus Christi as one of three schools—the others were Lincoln School of Teachers College and Evander Childs High School (whose principal, Dr. Hymen Alpern, was a leader in the Pan American movement)—as sites for Latin-Americanism, within the framework of the Good Neighbor Policy and Pan Americanism, a doctrine highly criticized in Latin America as a tool of imperialism.^115^

Of relevance is the connection Corpus Christi School built with assistant superintendent of New York Schools John J. Loftus, a Catholic who, before extending active methods to the New York City school system (what he called a conservative form of progressive education) visited Corpus Christi many times, bringing teachers to observe the classes. Loftus also enjoyed a close relationship with Ford.^116^ Corpus Christi School was also connected with large network organizations integrated by progressive-minded activists. For example, with the assistance of the Sisters, the school was a site for Reconciliation Trips, Inc., which organized visits, particularly to poor neighborhoods and minority groups, and also gave talks aimed at promoting multicultural awareness.^117^ Social Gospeler Bishop Francis J. McConnell, of the Methodist Episcopal Church, New York area, and Dorothy Day, from the *Catholic Worker*, were also board members of the extensive Reconciliation Trips, Inc.^118^

## Conclusion

As we have seen, although largely forgotten by historians, Corpus Christi School offers an important case of progressive education. The school was an expression not only of Catholic progressive education but also of a creative Catholic reading of John Dewey and William Kilpatrick’s educational theories and practices. Progressive/new education was not limited to Dewey and Kilpatrick, however, and was also not necessarily related to pragmatism, although Ford and the Sisters and their associates generated in practice an interpretation that adhered to Dewey’s central tenets but without dealing with its metaphysics. The school’s relationship with Teachers College was a special feature, as well as Father Ford’s involvement with human rights and ecumenical organizations of various sorts, an involvement that nourished his inclusive conception of schooling and education. Corpus Christi School had a good reputation, and Father Ford related that Professor Alexander, a member of the Teachers College faculty, said in 1939 “that the finest elementary school in the country was Corpus Christi across the street.”^119^

Ford had to contend with a politically and theologically conservative archdiocese at a time when, although the Vatican had worked out a conservative adaptation to modernity before World War II, there was an emerging movement outside toward a new approach to the relationship with the world and other religions conveyed in the *nouvelle théologie*, the precursor of Vatican II.^120^ (Despite all of the Vatican’s directives, one will not find uniformity in the Church.)

The Sisters’ familiarity with the project method was fundamental for Corpus Christi’s success. However, the school cannot be considered in isolation from the configurations crossing the religious and political spectrum: Protestant social gospel and its interdenominational approach, the New Deal era, the war, as well as conflicting views of citizenship formation. Education intersected with overlapping configurations. It was also a political tool and was seen as an agent for social change in one direction or another. Corpus Cristi’s close interconnection with Teachers College and Columbia University and the pedagogy that the Sisters developed and implemented, Ford’s familiarity with John Dewey’s work, his ecumenical vision of religion, his approach to education as a social transformative agency, and his revival of practices of the early Christian Church well before Vatican II all made Corpus Christi School an original, indeed avant-garde, Catholic school. It had a pluralist intercultural approach and Ford, deeply concerned with race relations and seeing racial issues as a structural problem, wanted to reduce racial tensions. Meanwhile, the school was inserted into a Catholic configuration that dissented from dominant hierarchical stands.

As a priest in a patriarchal institution, Father Ford had a wider field of action than did the Sisters, and his vision went beyond the project method and some of the progressive stands, which by and large were centered on tolerance. Judging from his presence in the *New York Times* and his participation on national committees, Father George Barry Ford was a public figure whose influence radiated well beyond his parish.

## Notes

1. Francis J. Ryan, “Monsignor John Bonner and Progressive Education in the Archdiocese of Philadelphia, 1925–1945,” *Records of the American Historic Society of Philadelphia* 102, no. 1/2 (1991): 17–43. Ellen Skerrett and Janet Welsh, “Reclaiming the Sinsinawa Dominicans’ Legacy of Catholic Progressive Education,” in *Preaching with Their Lives: Dominicans on Mission in the United States After 1850*, eds. Margaret M. McGuinness and Jeffrey M. Burns (New York: Fordham University Press, 2023), 65–99. For a comprehensive view of Catholic schools, see Ann Marie Ryan, *American Catholic Schools in the Twentieth Century* (Maryland: Newman and Littlefield, 2022).

2. The project method refers to purposeful activities in a social situation, working in a unit securing learning in an activity under the guidance of the teacher. William Heard Kilpatrick, “The Project Method,” *Teachers College Record* 19, no. 4 (1918): 319–36. C. A. Bowers, *The Progressive Educator and the Depression: The Radical Years* (Random House, 1969).

3. William Portier, “Catholic Intellectual Life,” in *The Cambridge Companion to American Catholicism*, eds. Margaret M. McGuinness and Thomas F. Rzeznik, (Cambridge University Press, 2021), 86–104, 97.

4. C. A. Bowers, The Progressive Educator and the Depression: The Radical Years (New York: Random House, 1969).

5. Lawrence A. Cremin, “The Transformation of the School,” *Progressivism in American Education, 1876–1957* (New York: Alfred A. Knopf, 1962), 181.

6. Quentin Skinner, “Meaning and Understanding in the History of Ideas,” *History and Theory* 8, no. 1 (1969): 3–53, http://www.jstor.org/stable/2504188; James Tully, “The Pen Is a Mighty Sword,” *British Journal of Political Science* 13, no. 4 (Oct. 1983): 489–509.

7. For example, there are substantive studies of the work of Leonard Covello (1887–1982), founder and principal of Benjamin Franklin High School in East Harlem, a community center school addressing the needs of Italian immigrants. Loren Krakowsky and Patrick Shannon, “Leonard Covello: A Study of Progressive Leadership and Community Empowerment,” *Occasional Paper Series* 24 (April 2010), DOI: https://doi.org/10.58295/2375-3668.1105; Michael Johanek and John Pucket, *Leonard Covello and the Making of Benjamin Franklin High School: Education as Citizenship Matters* (Philadelphia, PA: Temple University Press, 2007).

8. George B. Ford, *The Reminiscences of the Reverend George Barry Ford* (Oral History Research Office, 1956), 107; Microfiche A81, Ford, George, Columbia University Collection. https://dlc.library.columbia.edu/catalog/cul:r2280gb7q2/. George Barry Ford, *A Degree of Difference* (New York: Farrar, Straus & Giroux, 1969).

9. Ford, A Degree of Difference, 9.

10. Ibid., 21.

11. Ibid., 43.

12. See Ronald K. Goodenow, “The Progressive Educator, Race and Ethnicity in the Depression: An Overview,” *History of Education Quarterly* 15, no. 4 (1975): 365–94.

13. Ellen Skerrett and Janet Welsh, OP, “Reclaiming the Sinsinawa Dominicans’ Legacy of Catholic Progressive Education,” in *Preaching with Their Lives: Dominicans on Mission in the United States After 1850*, eds. Margaret M. McGuinness and Jeffrey M. Burns (New York: Fordham University Press, 2023), 65–99.

14. Ibid., 65–98.

15. Ibid., 86.

16. Catholic Theological Union, Dominican Sisters (OP), Sinsinawa, WI, https://ctu.edu/partners/dominican-sisters-o-p-sinsinawa-wi/, accessed January 5, 2024.

17. Ford, A Degree of Difference, 76.

18. Ibid., 17.

19. Lawrence Cremin, *American Education: The Metropolitan Experience* (New York: Harper and Row, 1988), 88.

20. Ford, A Degree of Difference, 69.

21. Roma Gans, “The New Education in Catholic Living,” *Religious Education: The Official Journal of the Religious Education Association* 33, no. 2 (1938): 93–99, 94. https://doi.org/10.1080/0034408380330205

22. Sister Mary Samuel, Superior, “To All the Sisters,” *Home*, November 16, 1936, p. 2, MSC Letter II.

23. See the New York City Landmarks Preservation Commission’s *Morningside Heights Historic District Designation Report*, February 21, 2017, http://s-media.nyc.gov/agencies/lpc/lp/2584.pdf. The 1930 Census, Manhattan (New York, New York county) was 87.4% White, 12% Black, and 6% “other.”

24. Ford, A Degree of Difference, 70.

25. Ibid.

26. “Church Dedication Is Led By Cardinal,” *New York Times*, October 26, 1936, 12.

27. Roma Gans, “The New Education,” 94. No other information was found.

28. Joan Smith, OP. Typed manuscript provided by archivist Cassie Vásquez, Dominican archives of Sinsinawa, Wisconsin; Ford, *A Degree of Difference*, 71.

29. Ford, A Degree of Difference, 71.

30. Ibid.

31. Smith, OP. Typed manuscript.

32. Ibid.

33. Sister Vivian to Mother Samuel, August 8, 1936, New York. GA-12, Box 1/7/021, provided by Cassie Vásquez.

34. Ibid.

35. *The Oath Against Modernism*, September 1910, https://franciscan-archive.org/bullarium/oath.html.

36. David Labaree, “Progressivism, Schools and Schools of Education: An American Romance,” *Paedagogica Historica* 41, nos. 1 & 2 (2005): 275–88.

37. For example, among many letters, see To Mother Samuel from George B. Ford, April 5, 1947. GA-12, Box 19/7/144.

38. Letter to Mother Samuel from George B. Ford, May 15, 1945, Corpus Christi Rectory, New York. Provided by Cassie Vásquez. GA-12, Box 19/7/135.

39. To Mother Samuel from George B. Ford, June 16, 1947. GA-12, Box 19/7/147.

40. For example, letters to Mother Samuel from George B. Ford, July 13, 1939; July 17, 1939; to Mother Samuel from George B. Ford, Corpus Christi Rectory, November 30, 1943. GA-12, Box 19/7/11b; letter (praising Sister Richardine, who was ending her term at the school) to Mother Samuel from George B. Ford, August 16, 1948. GA-12, Box 19/7/156. Manuscript letter praising Sister Vivian (who was ending her term as principal) to Sister Vivian from George B. Ford, July 12, 1942. GA-12, Box 19/7/099.

41. To Mother Samuel from Sister M. Bernadetta, Corpus Christi Convent, September 25, 1943, GA-12, Box 19/7/115A.

42. To Sister DeRicci from Sister Bernardetta, Corpus Christi School, September 22, 1943, GA-12, Box 19/114. Emphasis in the original. Ford considered Sister Bernadetta the kind of teacher the school did not need.

43. To George B. Ford from Mother Samuel, August 6, 1945. Note on the document provided by Cassie Vásquez. GA-12, Box 19/7/139.

44. Letter to Mother Samuel from George B. Ford, April 5, 1947, Corpus Christi Rectory, New York. Provided by Cassie Vásquez. Note on the letter: GA-12, Box 19/7/144.

45. For example, among many records, see Letter to Mother Samuel from George B. Ford, August 12, 1945, Corpus Christi Rectory, New York. Note on the document provided by Cassie Vásquez: GA-12, Box 19/7/140.

46. To Mother Mary Samuel from George B. Ford, Corpus Christi Rectory, New York, July 28, 1944. Written on the page: GA-12, Box 19/7/128A. Letter provided by Cassie Vásquez.

47. This appears in both articles by George Ford and Roma Gans. It is taken from “Tentative Plan for Guiding the Growth of Children in Corpus Christi School, 1937–38, pp. 9 and 10. See George M. Ford, Corpus Christi School, New York City, *Catholic Religious Education* 64 (May 1944): 537–39, 537; Gans, “The New Education,” 95.

48. Gonzalo Jover uses the expression “de-pragmatized” in “Democracy and Education Then and Now: The ‘De-Pragmatizing’ and ‘Ultra-Pragmatizing’ Readings of John Dewey’s Pedagogy,” in *Dewey in Our Time: Learning from John Dewey for Transcultural Practice*, eds. Peter Cunningham and Ruth Heilbronn (University College London, 2016), 40–56. A case in point is the doctoral thesis of Alberto Hurtado, S.J., at the Catholic University of Louvain.

49. Ford, A Degree of Difference, 222, 223.

50. Gans, “The New Education,” 95.

51. John Dewey, *Democracy and Education* (New York: Simon and Schuster, 1997).

52. Gans, “The New Education,” in particular 95.

53. Ibid., in particular 96.

54. Ibid., in particular 97.

55. Ibid.

56. “Teacher of the Eight Grades, Eighth Graders Examine Their Attitudes,” *Journal of Educational Sociology* 16, no. 6 (1943): 358–63, in particular 359.

57. Gans, “The New Education,” in particular 97.

58. Corpus Christi School. Compiled with the assistance of the students. Undated pamphlet.

59. Rosa Bruno-Jofré and Martín Schiralli, “Teaching History: A Discussion of Contemporary Challenges,” *Encounters on Education* 3 (Fall 2002): 117–127, 122.

60. Joseph Edward Rowan, “The Social Frontier (1934–43): Journal of Educational Criticism and Social Reconstruction” (PhD diss., Case Western Reserve University, 1969); C. A. Bowers, *The Progressive Educator and the Depression: The Radical Years* (New York: Random House, 1969), 90–130.

61. Patricia Albjerg Graham, *Progressive Education: From Arcady to Academe: A History of the Progressive Education Association, 1919–1955* (New York: Teachers College Press, 1967). Teachers College also housed conservative scholars such as Isaac Kandel and behaviorist psychologist Edward Thorndike.

62. However, the anti-Nazi corollary of the same year was written in German, addressed to the German people, and did not condemn Nazism as such. James Chappel, *Catholic Modern. The Challenge of Totalitarianism and the Remaking of the Church* (Harvard University Press, 2018), 63.

63. Ford, The Reminiscences, 107.

64. Ann Marie Ryan, American Catholic Schools in the Twentieth Century. Encounters with Public Education Policies, Practices, and Reforms (New York: Rowman & Littlefield, 2022), 83.

65. Ford, The Reminiscences, 75.

66. Ibid., 93.

67. Ibid.; Tomas A. Shannon, “Rerum Novarum,” in *Modern Catholic Social Teaching. Commentaries & Interpretations*, ed. Kenneth R. Himes, O.F.M. (Washington, DC: Georgetown University Press, 2011), 127–50.

68. Christine Firer, “Quadragesimo Anno,” in *Himes*, 151–74.

69. Kenneth R. Himes, O.F. M., ed. Modern Catholic Social Teaching. Commentaries & Interpretations.

70. James Chappel, Catholic Modern: The Challenge of Totalitarianism and the Remaking of the Church (Harvard University Press, 2018), 67.

71. Ford’s work in Harlem will be the topic of another paper. Harlem in the 1920s and 1930s was a center of political and cultural activism. The Harlem Race Riot of 1935 to protest police brutality is a landmark.

72. Letter to Mother Samuel from George B. Ford, Corpus Christi Rectory, March 16, 1936. GA-12, Box 12/7/047.

73. Letter to Mother Samuel from George B. Ford, Corpus Christi Rectory, May 20, 1942. GA-12, Box 19/7/090.

74. Ibid.

75. Ibid.

76. Ibid.

77. Letter from George B. Ford to Mother Samuel, Feast of Purification, n.d. (tentative date February 1948). GA-12, Box 19/7/153.

78. Because of increasing difficulties in 1939, Ford requested that he be relieved of his duties as counselor to 4,000 Catholic students at Columbia. This would include the chaplaincy of the half-dozen Newman Clubs. He was relieved of that chaplaincy as a result of remarks he had made at one of their monthly meetings at the Corpus Christi auditorium. Ford remained chaplain at Columbia until June 30, 1945. Club meetings now had speakers of an elementary catechetical sort. Ford, *A Degree of Difference*, 100. Ford was reprimanded on many occasions, and even forbidden from accepting an award and speaking in public.

79. Ford, A Degree of Difference, 108.

80. Ibid., 104.

81. Letter to Mother Samuel from George B. Ford, June 28, 1938, Corpus Christi Rectory, New York. GA-12, Box 19/7/51. Provided by the archivist of the congregation.

82. Ibid.

83. Ibid.

84. Letter from Mother Samuel to George B. Ford, July 14, 1938. GA-12, Box 19/7/053.

85. Letter to Mother Samuel from George B. Ford, Corpus Christi Rectory, New York, July 19, 1938, GA-12, Box 19/7/056.

86. Letter to Mother Samuel from George B. Ford, Corpus Christi Rectory, New York, July 19, 1938, GA-12, Box 19/7/057.

87. Letter to Mother Samuel from George B. Ford, Corpus Christi Rectory, New York, August 8, 1938, 1/060.

88. Letter to Mother Samuel from George B. Ford, Columbia University, Office of the Counsellor for Catholic Students, April 27, 1937. GA-12, Box 19/7/035.

89. Father George Ford, Corpus Christi School, New York City, *Catholic Religious Education* 64 (May 1944): 537–39, 537. Pius XI, “On Christian Education (*Divini Illius Magistri*)” (Libreria Editrice Vaticana, 1929), https://www.vatican.va/content/pius-xi/en/encyclicals/documents/hf_p-xi_enc_31121929_divini-illius-magistri.html

90. *Divini Illius Magistri* (1929), Encyclical of Pope Pius XI on Christian Education to the Patriarchs, Primates, Archbishops, Bishops, and Other Ordinaries in Peace and Communion with the Apostolic See and to All the Faithful of the Catholic World, article 15. https://www.vatican.va/content/pius-xi/en/encyclicals/documents/hf_p-xi_enc_31121929_divini-illius-magistri.html.

91. This reading was done in light of Quentin Skinner’s theory in his “Meaning and Understanding.”

92. Letter to Mother Samuel from George B. Ford, Corpus Christi Rectory, August 24, 1943. GA-12, Box 19/7/110.

93. Frances G. Sweeney, “A Catholic Progressive School,” *Commonweal* 28, no. 22 (Sept. 23, 1938): 548–50.

94. Sweeney, “A Catholic Progressive School,” 549.

95. Ibid.

96. Ibid., 550.

97. Ibid.

98. Clara C. Glenn, “Progress for Catholic Schools?” *Commonweal* 29, no. 2 (Nov. 4, 1938): 38–40.

99. Sister Mary de Lourdes, “Education and Escapism,” *Commonweal* 29, no. 14 (Jan. 27, 1939): 376–78.

100. Elizabeth Vos, “School News. Corpus Christi School,” *The Curved Horn* 2, no. 1 (Feb. 1937): 4. Published by Teachers College, Fordham University, New York.

101. For example, “Parochial School Here Looked Upon as a Model,” *The Catholic News* (July 16, 1938): 5–7; Jean Laurie Holzhauer, “N.Y. Parish School Tries Old-With-New Experiment to Make Better Christians and Citizens for Tomorrow,” *Catholic Herald Citizen* (Oct. 25, 1952): 13–14.

102. “The Corpus Christi School: Is It Rosminian or Progressive?” Anonymous typed page provided by the archivist with this note: “Taken from the *Educational Signpost* published by the American Education Association for February 1939.” The specialized librarian was unable to locate the supposed published version.

103. Letter from George Barry Ford to Mother Samuel, August 24, 1943, Dominican Archives, Sinsinawa, Wisconsin, Box 19/7/110.

104. Sister Mary Samuel, letter to all the Sisters, Rome, June 28, 1936. MSC Letter J. Provided by Cassie Vásquez.

105. Letter from George Barry Ford to Mother Samuel, August 24, 1943, Dominican Archives, Sinsinawa, Wisconsin, Box 19/7/110.

106. Ford, A Degree of Difference, 57.

107. Ibid., 91–92.

108. Shafali La, “1930s Multiculturalism: Rachel Davis DuBois and the Bureau for Intercultural Education,” *The Radical Teacher* 69 (May 2004), Progressive Education, 18–22. https://www.jstor.org/stable/20710241

109. Ford, A Degree of Difference, 140.

110. Ibid., 158–59. For the Carnegie Church Peace Union, see “Editorial Comment, The Carnegie Church Peace Union,” *The American Journal of International Law* 8, no. 2 (April 1914): 349–51.

111. Ford, *A Degree of Difference*, 158–59. “Rev. George B. Ford Declares Public Name Calling Divides Their Respective Faiths,” *New York Times*, Nov. 15, 1947, 15. ProQuest Historical Newspapers.

112. Ford, A Degree of Difference, 52

113. Ibid., 203.

114. Ibid., 116.

115. “Three Schools Here Become Centers of Inter-American Relations Teaching,” *New York Times*, March 1, 1942, D6; Proquest Historical Newspapers.

116. “Schools Extend Activity Program; Experiment Begun 5 Years Ago Is Found to Be in Practice in 25% of System,” *New York Times*, February 16, 1940, 19. To Mother Mary Samuel from John J. Loftus, Assistant Superintendent in Charge of Curriculum, Board of Education of the City of New York, June 17, 1942. AA-12, Box 19/7/096.

117. Letter to George B. Ford from Clarence V. Howell, Director Reconciliation Trips, Inc. Letter provided by Cassie Vásquez.

118. Ford was not involved in the Catholic Worker Movement.

119. Letter to Mother Samuel from George B. Ford, December 21, 1939, GA Box 19/7/069. In the same letter, Ford tells Mother Samuel that the principal of Horace Man School (Teachers College), who worked with the National Broadcasting Corporation to prepare teachers to use radio in education, brought his class to Corpus Christi with the Educational Director of the Corporation.

120. Jürgen Mettepenningen, Nouvelle Théologie. New Theologoy. Inheritor of Modernism, Precursor of Vatican II (T & T Clark, 2010).

